# Cyclosporine a 0.05% eye drops for the treatment of subepithelial infiltrates after epidemic keratoconjunctivitis

**DOI:** 10.1186/1471-2415-12-42

**Published:** 2012-08-18

**Authors:** Seydi Okumus, Erol Coskun, Mehmet Gurkan Tatar, Erdal Kaydu, Ramazan Yayuspayi, Aysegul Comez, Ibrahim Erbagci, Bulent Gurler

**Affiliations:** 1 University of Gaziantep, Faculty of Medicine, Department of Ophthalmology, Gaziantep, Turkey; 2 Nizip State Hospital, Department of Ophthalmology, Gaziantep, Turkey

**Keywords:** Epidemic keratoconjunctivitis, Corneal subepithelial infiltrate, Topical 0.05% cyclosporine A

## Abstract

**Background:**

To evaluate the treatment with topical 0.05% cyclosporine A (CsA) in patients with subepithelial corneal infiltrates (SEI).

**Methods:**

We reviewed 16 patients (22 eyes) before and after the treatment with 0.05% CsA eye drops. All patients had been treated previously with topical corticosteroids without any improvement and also they had to stop the medication secondary to intraocular pressure elevation. The objective data recorded included best-corrected visual acuity (BCVA), evaluation of corneal subepithelial infiltrate scores (CSIS), intraocular pressure (IOP) prior to treatment and the last follow-up visit.

**Results:**

Six males (37.5%) and 10 females (62.5%), mean age of 35.2 ± 16.6 years, were included. The patients’ average topical CsA use duration was 5.1 ± 3.5 months (1 – 13 months). The average follow up time of the patients was 9.2 ± 4.7 months (4 – 22 months). One patient, although he didn’t have a 0 scale of SCIS, did not show up for follow up examinations after six months. The mean BCVA (logarithm of the minimum angle of resolution) before and after the treatment were 0.15 ± 0.15 and 0.07 ± 0.07 respectively, CSIS 1.68 ± 0.89 and 0.23 ± 0.53 respectively, IOP 18.50 ± 3.82 and 16.86 ± 2.76 mmHg respectively. There were statistically significant improvements in BCVA (p = 0.002), reduction of CSIS (p = 0.002) and reduction of IOP (p < 0.001) prior to treatment and the last follow-up visit. 18 eyes (81.9%) showed clinical improvement and 4 (18.1%) had decreased SEI which did not fully disappear during the treatment period. The eyes which reached CSIS score 0 (18 eyes) were treated with CsA for 1 – 13 months; while the eyes which had clinical improvement but had not CSIS score 0 (4 eyes) were decided to discontinue of CsA treatment in last follow-up visit. There were recurrences in 2 eyes 3 months after the treatment. Patients reported reduction in the severity of symptoms after the treatment. Most of the patients reported no foreign body sensation, glare, or other side effects with topical CsA treatment. Overall, patients noted an improvement in vision and satisfaction with topical 0.05% CsA treatment.

**Conclusions:**

Topical 0.05% CsA is a safe and effective alternative treatment in patients with SEI who do not respond to other treatment modalities or have undesired side effects from topical steroids.

## Background

Epidemic keratoconjunctivitis (EKC) is one of the most common ocular viral infections caused by adenoviruses. Adenovirus family consists of different serotypes. Serotypes 8, 11 19 and 37 are held responsible for EKC and it is known that especially serotypes 8 and 19 cause epidemics [1,2]. Disease presents itself after an incubation period which usually takes 2 to 14 days. Mixed papillary and follicular response of the conjunctiva, eye pain, burning eyes, itching of the eyes, diffuse hyperemia, chemosis, serous discharge and ipsilateral periauricular lymphadenopathy can be observed during the course of the disease. In approximately 80% of the cases, keratitis in the form of diffuse superficial punctate keratitis, focal epithelial punctate keratitis, and subepithelial infiltrates (SEI) follow the conjunctivitis in a manner of 1 to 3 weeks. Subepithelial infiltrates are small, round and grayish lesions. They are composed of residues of antigen and lymphocyte accumulations adhered to surface stromal cells. The lesions disappear without causing scarring or neovascularization. They are usually bilateral and frequently asymmetrical. They may stay dormant in the cornea for months or years, or they may cause acute symptoms such as decrease in visual acuity, halo, glare, and photophobia [3,4]. Topical corticosteroids may suppress the symptoms and findings of EKC, however due to extended use of these agents problems such as cataract, glaucoma, and tendency to super infections may occur [5-7].

There are reports that show the efficacy of topical CsA (with concentrations of 1% and 2%) in the acute phase of adenoviral infections in the therapy of early local symptoms and in decreasing the incidence of corneal opacities and in the therapy of active subepithelial infiltrates during the chronic phase [3,8-10]. However, there are no detailed studies performed with topical 0.05% CsA in the therapy of corneal subepithelial infiltrates. The aim of this study was to assess the efficiency of topical 0.05% CsA (Restasis®, Allergan, Irvine, California, USA) in treating the corneal subepithelial infiltrates that have persisted for more than three months in patients with adenoviral epidemic keratoconjunctivitis.

## Methods

This study was performed on the 22 eyes of 16 patients who were referred to our clinics between the dates of September 2007 and October 2011, and who had subepithelial infiltrates following epidemic keratoconjunctivitis that persisted more than three months and who did not adequately respond to topical corticosteroids or in whom 3 of the corticosteroids were discontinued due to their side effects and 0.05% topical CsA were started. Age, gender, affected eyes, BCVA (logarithm of the minimum angle of resolution [LogMAR]) measurements prior to and after the treatment, IOP measurements (with non contact tonometers) were recorded and detailed biomicroscopic anterior segment examinations were performed. The corticosteroids used, average corticosteroid use, topical 0.05% CsA use durations and median follow up durations were recorded. Corneal subepithelial infiltrate scoring (CSIS) that varied between 0 and 4 were constituted according to the number of SEI seen in biomicroscopic examinations (0: no infiltrate, 1: 1–5, 2: 6 – 10, 3: 11 – 15, 4: more than 16 infiltrates) [11]. All patients were treated with topical 0.05% CsA (Restasis®) for a month as follows: 4 times a day of topical 0.05% CsA (Restasis®), in addition to the topical corticosteroids they were using for the first 15 days, and then 2 times a day of topical 0.05% CsA (Restasis®) after topical corticosteroids were discontinued. At the end of the first month, treatment was discontinued in those patients with a CSIS of 0 and monitorization of these patients began. In the patients with CSIS other than 0 were continued on topical 0.05% CsA (Restasis®) therapy either once a day or once in every other day according to the symptom intensity and clinical examination results.

All analysis were carried out by SPSS 17.0 (Statistical Package for Social Sciences Inc. Chicago, IL, USA) software. Wilcoxon signed rank test was used for statistical analysis and values of p < 0.05 were considered to be significant. Gaziantep University Ethics Committee approval and informed consent form from the participants were attained.

## Results

Twenty-two eyes of sixteen patients were included in the study, of these 6 (37.5%) were male and 10 (62.5%) were female. The average age of the patients was 35.2 ± 16.6 years (13 – 75 years). Subepithelial infiltrates were located in the right eye in 14 patients whereas 8 patients had them in the left eye. Prior to treatment, BCVA was 0.00 LogMAR in 10 eyes and there were varying degrees of visual loss in 12 eyes with values of 0.40 – 0.10 logMAR. Prior to therapy, average intraocular pressures were recorded to be 18.50 ± 3.82 mmHg (11 – 26 mmHg), and average CSIS scores were 1.68 ± 0.89 (Table 1). The average duration of corticosteroid use of the patients was 6.7 ± 3.9 months (3–14 months). Fluorometholone therapy was performed on eleven eyes, Loteprednol etabonate on nine eyes and Prednisolone asetate on two eyes. Before receiving topical CsA therapy, 8 eyes of 7 patients were on anti - glaucomatous therapy. After the first month, CSIS scale was found to be 0 in all eight eyes. In these patients, the treatment was discontinued and follow up of the patients began after this time interval. There was no recurrence during the follow up period in eight eyes. In 14 eyes, topical 0.05% CsA treatment of one per day or one every other day was continued depending on the subjective symptoms of the patients (halo, glare and photophobia etc.) and their examination findings (BCVA, CSIS) (Table 1). In one eye with a CSIS scale of 2, in two eyes with a CSIS scale of 3 and one eye with a CSIS scale of 4 at the start, scale did not revert to 0 in spite of the treatment. The average topical CsA use duration of the patients was 5.1 ± 3.5 months (1–13 months) (Table 1). The average follow up time of the patients was 9.2 ± 4.7 months (4 – 22 months). One patient, though not having a 0 scale of SCIS, did not show up for follow up examinations after six months. In the last control, the average BCVA was recorded to be 0.07 ± 0.07, whereas IOP was 16.86 ± 2.76 mmHg and CSIS was 0. 23 ± 0.53, respectively. When the average BCVA, CSIS and IOP values prior to the therapy and in the last control were compared, there was a statistically significant difference among them [(p = 0.002), (p = 0.002), (p < 0.001), respectively] (Table 2). In the last control exam, symptoms such as glare, photophobia and discomfort in the eye that were present in 18 eyes (81.8%) were completely resolved. In 4 eyes (18.2%), which still had subepithelial infiltrates, photophobia and glare were still present. There were no eyes on anti-glaucomatous therapy during the last control examinations. The average after discontinuation of medicine treatment was in the period of 4.1 ± 2.6 months (0–12 months), at the end of the first month of CsA discontinuation no recurrence was observed in eight eyes however three months after the discontinuation of medication recurrence was observed in two eyes which had CsA medication for 7 and 8 months respectively (Table 1). In 16 of the 18 eyes which were free of subepithelial infiltrates had no recurrences.

**Table 1:** Clinical course of patients, in whom SEI developed after EKC, that were treated with topical 0.05% CSA eye drops

**Patient**	**Age/Sex**	**Affected eye**	**Initial BCVA (LogMAR)**	**Initial IOP (mmHg)**	**Initial CSIS**	**Duration of follow up with Corticosteroids Therapy (month)**	**CsA treatment for increased ocular pressure (Yes/No)**	**Duration of follow up with 0.05% CSA Therapy (month)**	**BCVA at last follow-up visit**	**IOP at last follow-up visit**	**CSIS at last follow-up visit**	**The length of follow up without CsA (month)**	**Recurrence after CsA treatment (Yes/No)**
1	13/F	RE	0.00	11	1	12	Y	1	0.00	11	0	6	N
2	38/F	LE	0.00	14	1	9	N	6	0.00	12	0	5	N
3	26/F	RE/LE	0.10**/**0.22	16/18	1/2	3/5	N	1/1	0.00/0.10	14/19	0/0	4/3	N
4	47/F	RE	0.00	15	1	3	N	7	0.00	17	0	2	N
5	40/F	RE	0.22	25	1	3	Y	1	0.00	20	0	4	N
6	30/M	RE/LE	0.40**/**0.00	26/22	4/1	4/7	Y	13/8	0.20/0.00	21/19	2/0	5/3	N
7	18/M	RE	0.40	23	3	6	Y	7	0.15	20	1	2	N
8	50/M	RE	0.22	18	2	7	N	6	0.10	16	1	0	N
9	27/M	RE/LE	0.00**/**0.30	15/17	1/3	8/9	N	1/1	0.00/0.15	13/16	0/0	3/3	N
10	40/F	LE	0.00	19	2	4	N	7	0.00	19	0	3	Y
11	16/M	RE	0.00	17	1	3	N	6	0.00	14	0	4	N
12	32/F	RE	0.20	16	1	3	N	1	0.10	17	0	3	N
13	75/M	RE/LE	0.00**/**0.20	15/22	1/2	5/4	Y	7/8	0.00**/**0.15	16/19	0/0	4/3	Y
14	60/F	RE	0.00	20	1	11	Y	6	0.00	19	0	10	N
15	30/F	RE/LE	0.40**/**0.30	17/21	3/2	13/8	N	10/8	0.20/0.10	15/18	1/0	12/3	N
16	22/F	RE/LE	0.00**/**0.30	17/23	1/2	14/10	Y	1/5	0.00**/**0.10	17/19	0/0	6/4	N

*F*: Female, *M*: Male, *RE*: Right eye, *LE*: Left eye, *Y*: Yes, *N*:No, *BCVA*: Best corrected visual acuity, *IOP*: Intraocular pressure, *CSIS*: Corneal subepithelial infiltrate scores.

**Table 2:** Patient data before and after treatment with 0.05% CsA

**Patient data**	**Before CsA treatment**	**After CsA treatment**	***p***
BCVA	0.15 ± 0.15	0.06 ± 0.07	0.002
IOP (mmHg)	18.50 ± 3.83	16.86 ± 2.76	0.002
CSIS	1.68 ± 0.89	0. 23 ± 0.53	<0.001

***BCVA***: Best corrected visual acuity (LogMAR), ***IOP*:** Intraocular pressure, ***CSIS***: Corneal subepithelial infiltrate scores, data are shown Mean **± SD**, Statistical analysis for used Wilcoxon Signed Ranks test, p values less than 0.05 are reported as statistically significant.

## Discussion

Epidemic keratoconjunctivitis (EKC) can present with symptoms such as chemosis, eye pain, itching, hyperemia, photophobia and swelling of eyelids that can have a negative effect on everyday life. In approximately 80% of the patients, keratitis with subepithelial infiltrations (SEI) can ensue the conjunctivitis [3,4]. SEI can result in a decrease of visual acuity, halo, irregular astigmatism and photophobia. Some studies report that the decrease in visual acuity caused by SEI can resolve in a matter of weeks and that it can persist for years in some cases [1,10]. In ocular adenovirus infections, virus static agents; such as trifluridine, vidarabine and ganciclovir; were tried but none were found to be effective in treatment [12-14]. It is reported that long term corticosteroid use in adenovirus infections are effective but can cause cataracts, glaucoma and super infections [5-7].

Our study group consisted of patients who had no regression in SEI in spite of corticosteroid use of 3–14 months (average 6.7 ± 3.9 months) and those who had to stop the corticosteroids because of intraocular pressure due to 5 long term use. There was enough intraocular pressure to cause varying visual loss in twelve eyes with values of 0.40 – 0.10 logMAR and anti glaucomatous therapy to start in 8 eyes.

It is reported that topical CsA plays a role in the inhibition of T lymphocyte proliferation and activation and suppression of the inflammation of the ocular surface lacrimal gland [15]. It is reported in the literature that topical CsA is effective in various concentrations in ocular inflammation cases such as vernal keratoconjunctivitis, ulcerative keratitis due to rheumatoid artritis, Thygeson superficial punctate keratitis, anterior uveitis, corneal graft rejection, superior limbic keratoconjunctivitis, greft versus host disease, micotic keratitis, Cogan syndrome, Behçet’s disease, herpetic stromal keratitis, Mooren ulcer and atopic keratoconjunctivitis [16-30]. Topical 0.05% CsA was used in the treatment of dry eye syndrome, in minimizing the risk of recurrence after pterygium surgery, in treatment of ocular graft-versus-host disease and in the treatment of meibomian gland dysfunction successfully without any systemic or ocular side effects [31-35]. In our study, there were improvements in the signs and symptoms caused by SEIs, that developed after EKC infections, by using topical 0.05% CsA and there were no ocular or systemic side effects observed.

In studies conducted by using topical CsA in the treatment of acute and chronic adenovirus infections, it was reported that CsA was effective in concentrations of 1% and 2% and that the SEI’s were completely obliterated or majorly reduced after a 3 to 4 week therapy [1,3,8-10,16]. After a one month topical 0.05% CsA therapy, it was observed that in 8 (36.3%) eyes SEIs were completely cleared, and in 10 eyes (45.45%) this result was achieved in the last follow up visit. In remaining 4 eyes (18.2%) they were not completely cleared but decreased in number. The treatment was discontinued in the eyes with no SEI left, and it was continued in doses of once a day or once in every other day in eyes which still had SEIs.

Jeng and colleagues have reported that a single dose per day or every other day of 1% or 0.05% topical CsA treatment following an initial therapy of topical 1% CsA and steroids for a month was effective in SEI treatment [9]. The average topical 0.05% CsA use of the patients was 5.1 ± 3.5 months (1–13 months), and the follow up time was 9.2 ± 4.7 months (4–22 months). The increase in the visual acuity, decrease in CSIS score and the intraocular pressure in the last follow up visit was significant. There were no patients on antiglaucomatous therapy. In the last follow up visit, 18 eyes out of 22 (81.8%) had a CSIS score of 0, and 4 (18.2%) eyes had a decrease in SEI numbers. There were 2 recurrences out of 18 eyes (11%) after the therapy was discontinued.

Reinhart and colleagues have reported that there were amelioration and decrease in SEI number in the 48 eyes out of 70 which had SEIs after EKC infection after a therapy with 2% topical CsA, and there were no recurrences after therapy was discontinued [10]. In the literature, it has been reported that 1% topical CsA was tolerated well and did not cause any systemic side effects in long term follow ups [8,9]. Romanowski and colleagues reported that in their trials 0.5% and 2% topical CsA treatments were effective in decreasing the number of SEI formations, however it was claimed that this agent could facilitate the risk of endemics by increasing viral replication [11].

When the studies in the literature were analyzed, it was observed that topical CsA of varying concentrations between 0.5-2% were used in the inhibition of T lymphocyte proliferation and activation and in eradicating the symptoms and minimizing the recurrences of SEI in acute adenoviral infections. In chronic phases, it is reported to be an effective and safe treatment of SEIs and minimizing the recurrence risk. However, there were no detailed studies concerning the corneal subepithelial infiltration therapy with lesser concentrations of topical 0.05% CsA.

In this study, we had two recurrent cases (11.12%) who were treated with topical CsA for 4 and 8 months. While the average treatment time for all eyes was 5.1 ± 3.5 months. After 9.2 ± 4.7 months (4 – 22 months) of treatment, only 2 eyes (recurrence in two eyes was seen in 3rd month) out of 18 were detected as recurrent. In 16 of the 18 eyes, which were free of subepithelial infiltrates, had no recurrences. As for our experience, treatment of SEI by using CsA should be continued until the CSIS is seen as 0. However, whether SEI will recur after treatment or when will it recur cannot be foreseen. Therefore, follow up visit duration should be as longer as possible. Further studies regarding the ideal follow up visit duration after treatment with CsA is warranted. The small number of the patients, the absence of control group, and the retrospective design of the study seems to be the limitations of this study. For treatment of SEI development after EKC, two different treatment methods are used which were proven to be efficient are topical korticosteroid and topical CsA. Because the patient group was consisted of patients who did not benefit from at least 3 months of treatment with topical corticosteroids or who develop steroid-related side effects; current treatment could not be continued. Because the clinical symptoms of patients affect their daily life, control group who will be treated with placebo could not be constructed. However, the difficulty of building control group for the cases with subepithelial infiltrates who do not exert improvement in symptoms and findings after 3-month treatment with corticosteroids should be taken into consideration. On the other hand, clinician must choose and apply the most reliable treatment strategy which resolve patient complaints and improves clinical findings for SEI. Since our data were acquired without a control group, we cannot resolve the possibility of spontaneous remission of SEI as the natural history of the disease in this study.

A masked controlled study of topical 0.05% CsA in human subjects with larger patient populations could better define the natural history and effect of treatment of this disorder. As an alternative approach, different concentrations of CsA can be compared using different treatment groups for prospective studies.

## Conclusions

As a result, the authors claim that topical 0.05% CsA is a safe and effective option in eradicating the symptoms of adenovirus infections and in the therapy of cases which have steroid resistant SEIs after chronic adenovirus infections.

## Competing interests

The authors declare that they have no competing interests.

## Authors’ contributions

SO conceived and designed the study. EC, MGT and IE monitored data collection. SO and RY performed analysis of the data and drafted the manuscript. EK, AC and BG contributed to the review of the manuscript. All authors read and approved the final manuscript.

## Author disclosure statement

The authors have no commercial associations or sources of support that might pose a conflict of interest. The authors declare that this study is not funded by any foundation or organization.

## Pre-publication history

The pre-publication history for this paper can be accessed here:

http://www.biomedcentral.com/1471-2415/12/42/prepub
